# An Evaluation of Mental Health Professionals’ Confidence in Performing Perinatal Assessments & The Need for the Development of an Assessment Tool

**DOI:** 10.1192/j.eurpsy.2022.2204

**Published:** 2022-09-01

**Authors:** A. Delvi, L. Blake, A. Lapraik, G. Lewis

**Affiliations:** South London & Maudsley NHS Foundation Trust, Channi Kumar Mother & Baby Unit, Beckenham, United Kingdom

**Keywords:** assessment tool, Stafford Interview, women’s mental health, Perinatal psychiatry

## Abstract

**Introduction:**

Clinicians often do not have experience assessing perinatal patients unless they work as part of a perinatal team. Informal feedback points to a lack of confidence in performing perinatal assessments.

**Objectives:**

The aim of the project was to assess clinicians’ confidence in performing perinatal assessments in outpatient and inpatient settings including the Emergency Department. Additionally, we wanted to assess whether access to a perinatal assessment tool was beneficial. We hypothesise that clinicians lack confidence in performing perinatal assessments and would benefit from using a perinatal assessment tool.

**Methods:**

We designed a survey of 10 questions assessing the above. The survey was sent out to psychiatric trainees and nurses at South London & Maudsley NHS Foundation Trust. The participant’s confidence in completing perinatal assessments in various settings was assessed using a 5 point Likert scale.

**Results:**

52 responses were received. 50% of participants felt *not so confident* in performing perinatal assessments in the outpatient setting. 40.38%(n=21) of participants felt *not so confident* in exploring the mother and foetal relationship. 71.15% (n=37) of participants felt that they would benefit from additional teaching with 48.1% of participants citing that they would benefit from access to an assessment tool.

**Conclusions:**

As predicted, the results of the survey show that clinicians lack confidence in performing perinatal assessments. Therefore, we have commenced work on modifying the existing Stafford Interview. This is a structured interview that explores the obstetric and psycho-social background and psychiatric complications of pregnancy. The survey is due to be replicated in other project locations to allow transcultural comparison.

**Disclosure:**

No significant relationships.

